# Connecting Synaptic Activity with Plasticity-Related Gene Expression: From Molecular Mechanisms to Neurological Disorders

**DOI:** 10.1155/2016/7149527

**Published:** 2016-03-20

**Authors:** Pablo Muñoz, Armaz Aschrafi, Pablo R. Moya

**Affiliations:** ^1^Department of Pathology and Physiology, School of Medicine, Faculty of Medicine, University of Valparaíso, 2341386 Valparaíso, Chile; ^2^Interdisciplinary Center for Innovation in Health (CIIS), University of Valparaíso, 8380492 Valparaíso, Chile; ^3^Laboratory of Molecular Biology, National Institute of Mental Health, National Institutes of Health, Bethesda, MD 20819, USA; ^4^Nucleo Milenio en Biología de Enfermedades Neurosiquiátricas nuMIND, Instituto de Fisiología, Facultad de Ciencias, Universidad de Valparaíso, 2360102 Valparaíso, Chile

One of the most enticing endeavors in neurobiology is to examine how synapse activation leads to the expression of functionally important genes, which contribute to the maintenance and modulation of synaptic plasticity over time. To make these changes persistent, the implementation of various genetic and epigenetic mechanisms that collectively orchestrate the expression of synaptically relevant genes is critical. These findings are most gratifying as they provide an enlarging body of evidence that molecular mechanisms involving transcriptional and posttranscriptional regulation of synaptically relevant gene expression are critical for brain development, function, and neuronal plasticity. However, there is still a paucity of information on how specific molecular changes are regulated, how specific genes interact with each other during memory formation, and ultimately how these changes manifest functionally at the cellular and circuit levels. In this special issue, O. Khalaf and J. Gräff provided a comprehensive review on the mechanisms mediating the transformation from an unstable memory to a lasting one, including neural circuits and subpopulations of cells that can be recruited into a memory trace, as well as the structural changes occurring at synapses during memory formation.

We still have very limited understanding of the precise molecular mechanisms underlying the modulation of synaptic strength; over the past years a fragmentary picture is emerging through the identification of molecules whose loss of function impairs the experimental expression of synaptic plasticity. Some of these recently discovered molecules are highlighted by the authors of this special issue, representing a variety of classes of cellular functions ranging from transcription, translation, neurogenesis, gliogenesis, differentiation, Excitation/Inhibition (E/I) balance, trophic signaling, endocytosis, and neuritogenesis to synaptogenesis. Importantly, this special issue raises a series of novel hypotheses, on how single molecules contribute to precise cellular processes and how neurons collectively contribute to functional circuits, which are necessary and required for cognition. One of these hypotheses is raised by C. Engelmann and R. Haenold; in their inquiry of the transcriptional regulation of synaptic plasticity through NF-*κ*B, they provide compelling evidence that this transcription factor may be activated synaptically, since all the machinery required for the local activation of NF-*κ*B is concentrated in the postsynaptic density, and once activated, NF-*κ*B is translocated to the nucleus by a cytoskeletal dependent process [1] and calcium increase [2]. This mechanism would restrict the NF-*κ*B-dependent gene transcription only to stimulated neurons. In the case that stimulated neuron corresponds to a GABAergic neuron, NF-*κ*B regulates the expression of the GABA synthetizing enzyme glutamate decarboxylase 65 (GAD65), by modifying the E/I balance [3]. In the same line, the article by M. O. Caracci et al. points toward the participation of Wnt cascade, another important signaling pathway for synaptic plasticity and E/I balance. Interestingly, these authors describe alterations in both Wnt pathway and excitatory/inhibitory transmission in the etiology of autism spectrum disorders (ASDs); notably a high comorbidity of epilepsy in humans affected by ASDs is the most robust evidence of this relationship [4]. Consistent with the importance of E/I balance in epilepsy, M. Fuenzalida and C. Bonansco present a comprehensive review of the glutamatergic and GABAergic components of epilepsy but also bring to the discussion another key element involved in the synaptic function, the astrocyte, which has a major role in synaptic transmission, in what has been called the synapse tripartite [5].

Higher proportions of glial cells to neurons have been also found in both human brains and animal models of Down syndrome, another disorder with cognitive deficits. Changes in the generation rate of glial cells, therefore, might underlie such alterations in the E/I balance linked to this syndrome [6, 7]. In this regard, the article by H.-C. Lee et al. deepens on the differentiation of astrocytes, reviewing signaling pathway JAK/STAT, which is critical for gliogenesis [8] and is dysregulated in Down syndrome. From a mechanistic point of view, the STAT transcriptional activator must be phosphorylated and translocated to the nucleus to trigger gene expression required to initiate astrogliogenesis [9]. In contrast, Ngn1 activation promotes neurogenesis by competition with STAT by the binding to the coactivator p300/CBP. Complementing the topic of neurogenesis, the article by R. Ramírez-Barrantes et al. provides an overview of the involvement of the family of transient receptor potential V1 (TRPV1) in neurogenesis from neural precursors, along with cover aspects most studied in plasticity synaptic and neuronal excitability [10].

Knowledge of the molecular mechanisms that enable such activity-dependent expression of plasticity-related genes enhance our understanding of mechanisms attenuated in neurological diseases, thus opening the possibility of unraveling novel therapeutic targets to restore normal neuronal function.

In this regard, this special issue also encompasses the effect of some pathological and physiological conditions on neuronal function. P. Espinosa et al. examined the effect of neonatal exposure to sex hormones in the programming of dopaminergic neurons resulting in increased expression of tyrosine hydroxylase in ventral tegmental area and substantia nigra. On the other hand, D. A. Smagin et al. studied the effect of chronic social defeat stress in ribosomal gene expression. The authors found the greatest transcriptional effects in the hypothalamus and hippocampus, suggesting that these regions are more sensitive to stress.

Not surprisingly, in Alzheimer's disease, as well as in animal models of this disease, the hippocampus is also affected. In particular, in the article by B. Seifert et al., they elegantly demonstrated that the amyloid-beta peptide causes a dysfunction in vesicular transport of brain derived neurotrophic factor in transgenic mouse models of Alzheimer's disease. This alteration induced by amyloid-beta peptide could be due to changes in the expression of proteins involving vesicular movement and endocytosis such as Endophilins, as might suggest the study by J. Zhang et al., which showed that Endophilin 1 and 2 isoforms exhibit differential roles in synaptic vesicle endocytosis.

Understanding the precise interaction of synaptically relevant transcriptional regulation can have therapeutic potential for treatments and possibly improving these conditions, and the work by P. Lobos et al. fits within this premise: since it is known that oxidative stress contributes to the development of Alzheimer's disease, they studied the effect of astaxanthin, an antioxidant having free access to the brain, on the aberrant calcium signaling and decreased expression of type-2 ryanodine receptors (RyR2) induced by amyloid-*β* peptide oligomers (A*β*Os). They report here that astaxanthin protects hippocampal neurons in culture from the harmful effects of A*β*Os, preventing the generation of reactive oxygen species, activation of the transcription factor NFATc4, and RyR2 gene expression downregulation. Another example of how knowledge of the transcriptional mechanisms involved in neuronal plasticity can lead to improvement of certain conditions is the work of G. Sun et al. In this work it was found that three-dimensional cultures of spiral ganglion neurons using a combination of Matrigel and neurotrophic factors protect the culture of apoptosis and better preserve neuritic structures.

Collectively, this special issue highlights the mechanistic importance of synaptically relevant transcriptional regulation, which might open novel avenues for therapeutical approaches.
